# Pediatric Anti-N-methyl-D-aspartate Receptor Encephalitis Mimicking Glutaric Aciduria Type 1: A Case Report

**DOI:** 10.3389/fneur.2020.587324

**Published:** 2020-10-26

**Authors:** Daniel Almeida do Valle, Mara Lúcia Schmitz Ferreira Santos, Michelle Silva Zeny, Mara L. Cordeiro

**Affiliations:** ^1^Department of Neurology, Children's Hospital Pequeno Príncipe, Curitiba, Brazil; ^2^Faculdades Pequeno Principe, Curitiba, Brazil; ^3^Research Institute Pelé Pequeno Príncipe, Curitiba, Brazil; ^4^Department of Psychiatry and Biobehavioral Sciences, University of California, Los Angeles, Los Angeles, LA, United States

**Keywords:** anti-N-methyl-D-aspartate receptor encephalitis, glutaric acidemia I, dystonia, child, antibody

## Abstract

Anti-N-methyl-D-aspartate receptor (NMDAR) encephalitis is an immune-mediated disease that induces a wide spectrum of symptoms, especially in toddlers. These include acute-onset movement disorders, with neurological regression, and other associated neurological symptoms. Anti-NMDAR encephalitis remains a diagnostic challenge, especially in toddlers, with better prognosis associated with early treatment. We report the case of a 15-months-old boy who initially presented with vomiting and later with acute-onset dystonia after the administration of antiemetics. Within 14 days, the patient developed neuropsychomotor developmental regression and worsening dystonia. After ruling out an acute dystonic reaction and glutaric acidemia type 1 (GA-1), a final diagnosis of anti-NMDAR encephalitis was made. The patient responded well to immunomodulatory therapy. The present case underscores the importance of early treatment for patient prognosis and of including anti-NMDAR encephalitis in the differential diagnosis of acute-onset movement disorders.

## Introduction

Anti-N-methyl-D-aspartate receptor (NMDAR) encephalitis is an immune-mediated disease that induces a wide spectrum of symptoms. In suspected cases of anti-NMDAR encephalitis, the diagnosis can be confirmed with an anti-GluN1 immunoglobulin (Ig) C test (1). To highlight the importance of suspecting anti-NMDAR encephalitis when a patient presents with acute-onset movement disorders, we documented the case of a pediatric patient who was diagnosed with anti-NMDAR encephalitis after presenting with symptoms suggestive of glutaric aciduria type 1 (GA-1).

## Patient Information

A 15-months-old boy was admitted to our emergency department due to an episode of constipation and vomiting. Besides fever, the patient had no other evidence of recent infectious

disease and no history of previous hospitalization. Following an uncomplicated pregnancy and delivery, he was born to non-consanguine parents with no family history of neuropsychiatric disorders. The child had macrocephaly, which became apparent at 6 months of age.

The emergency department physician administered alizapride (1.25 mg/kg) as a form of antiemetic treatment. Subsequently, the patient developed orofacial dyskinesias, opisthotonos, limb dystonia, and chorea. These outcomes were suspected to be signs of an extrapyramidal-triggered reaction to alizapride. The patient was administered biperiden (0.05 mg/kg), which did not alleviate his abnormal movements. Furthermore, he soon developed behavioral arrest with ocular deviation and generalized limb hypertonia, which was consistent with a self-limited epileptic seizure that lasted for <1 min.

## Clinical Findings

**Figure 1:**
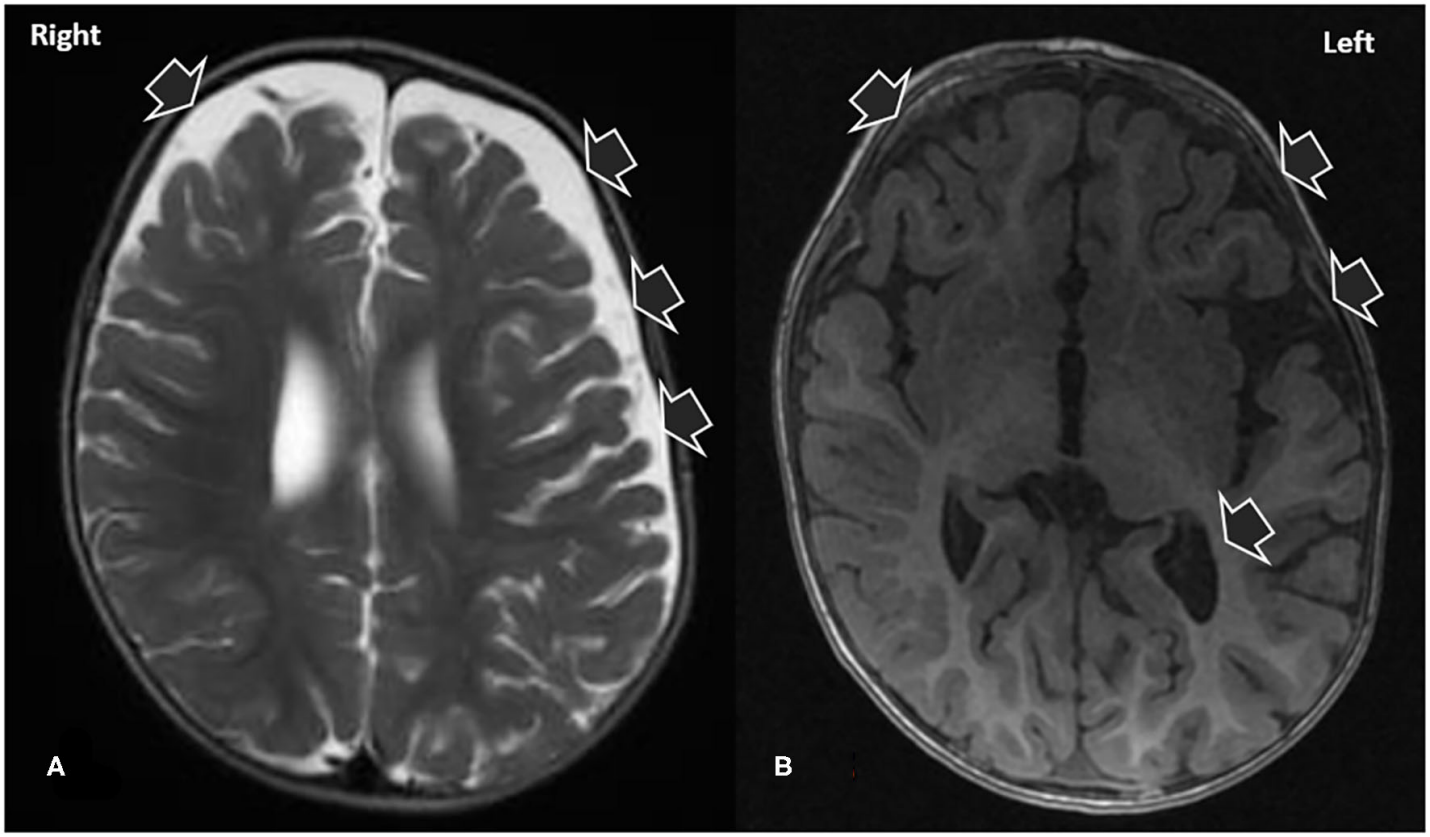
T2 **(A)** and T1 **(B)** magnetic resonance images demonstrating signs of craniofacial disproportionality and signs consistent with macrocrania. Note that the patient had an overall normal morphology, excepting the prominence of the ventricular system and CSF-filled spaces in the left cerebral convexity.

The patient was admitted to our hospital. The patient's general state worsened during the first 5 days of admission with the emergence of fever, irritability, agitation, and the regression of his neuropsychomotor development. Electroencephalography showed normal base activity, with occasional slow frequency rhythm in posterior regions. Epileptiform activity was not observed. With the exception of a small increase in lactate (2.4 mg/mL), no further metabolic changes were identified. The first cerebrospinal fluid examination, collected on the second day of hospitalization, presented white blood cells 4.37/mm3, glucose level of 60 mg/dL (glucose ratio of 0.77) and protein level of 17 mg/dL. Viral profile in CSF and normal blood for enterovirus, cytomegalovirus, Epstein Barr virus, herpes virus type I and II. As shown in Figure 1, brain magnetic resonance imaging (MRI) with contrast revealed areas of hypointensity in the frontotemporal regions of the brain.

## Diagnostic Assessment and Therapeutic Intervention

Considering the patient's history of acquired macrocephaly, our observations of bitemporal atrophy and the onset of developmental regression and movement disorder after the patient's febrile event informed our suspicion of glutaric aciduria type 1 (GA-1). The patient was treated with intravenously administered glucose and a hypoproteic diet. Despite therapy, the patient persists with significant generalized dyskinesia, aggravated by movement and sounds, remaining responsive to visual and sound stimuli. On day 5 of hospitalization he presented a dystonic status, with fever, profuse sweating, dystonia and hyperCKnemia (CK: 1,326 mg/dL). He was sedated with continuous midazolam. Treatment with baclofen, tizanidine, clonazepam, triexifenidil, and carbamazepine started in order to control dystonia. Progressive midazolam withdrawal was performed, with discharge from the Intensive Care Unit (ICU) after 14 days. After 29 days, results of metabolic and genetic testing for GA-1 were normal.

Given the normal whole-exome sequencing results, alternative diagnosis was considered, and the possibility of anti-NMDAR encephalitis suspected due to the acute onset of developmental regression, abnormal movements, seizures and altered mental status. In line with our suspicions, treatment with intravenous immunoglobulin (IVIg) (400 mg/kg/d) and intravenous methylprednisolone (30 mg/kg/d) for 5 days was then initiated. Within the first 2 days of IVIg therapy, the patient's abnormal movements improved markedly, and his cerebrospinal fluid (CSF) sample tested positive for IgG anti-GluN1 antibodies. Other antibodies related to autoimmune encephalitis were negative in CSF and serum. Malignancy workout was performed, finding no abnormalities. Upon the conclusion of the initial 5-days IVIg therapy, the patient was treated with the immunosuppressant azathioprine (4 mg/kg/d) and monthly IVIg infusions. The patient showed progressive improvement of his muscle tone, control of his dystonia, and the recovery of some developmental milestones.

## Follow-Up and Outcomes

**Figure 2:**
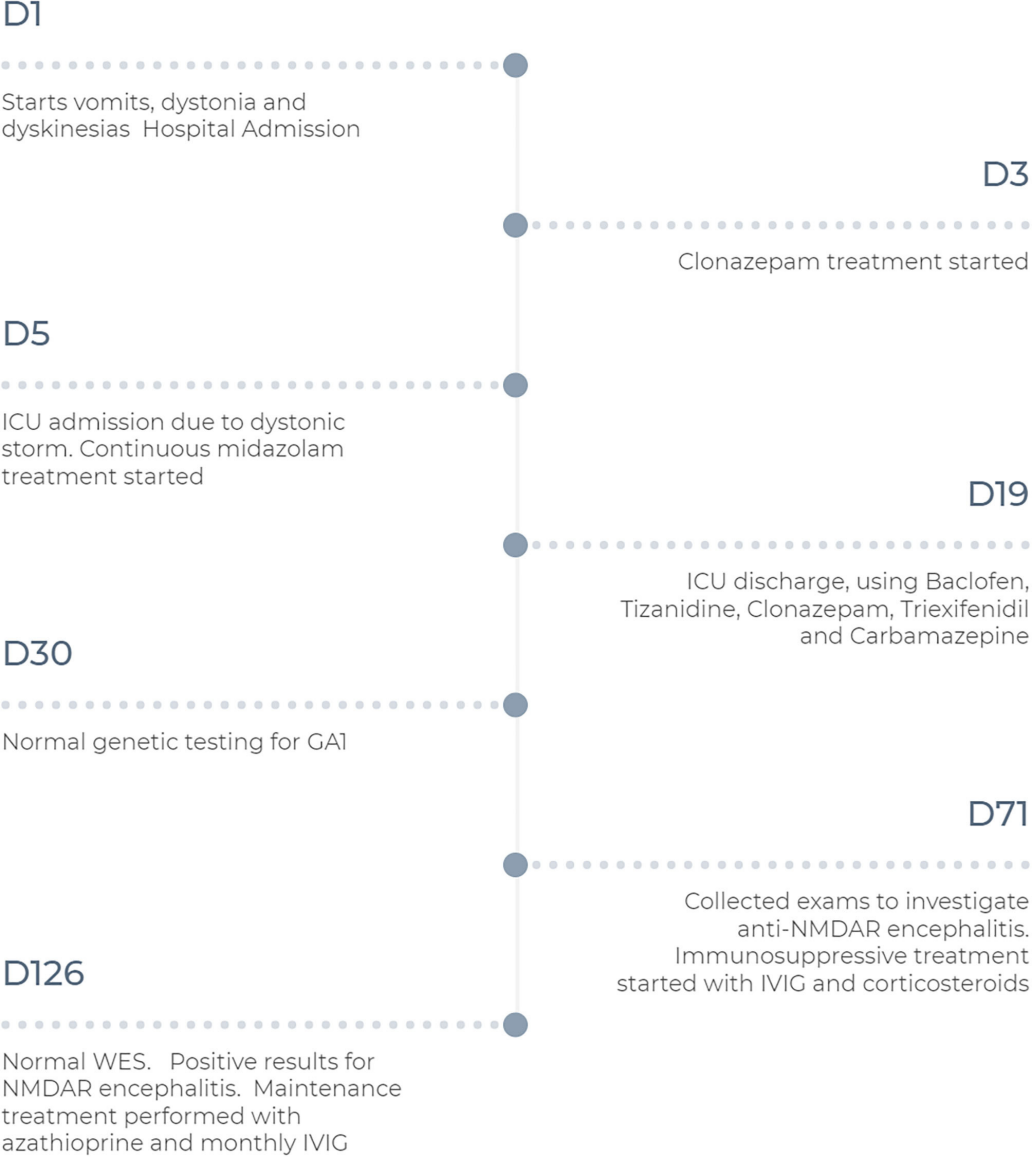
Timeline with treatment progression and diagnosis time. D, day; GA1, glutaric aciduria type 1; ICU, Intensive Care Unit; IVIG, intravenous immunoglobulin; NMDAR, Anti-N-methyl-D-aspartate receptor; WES, Whole Exome Sequence.

In the year following the patient's discharge from our hospital, the regimen of azathioprine and IVIg has been maintained, and the patient's development and control of dystonia have continued to improve. However, the patient maintains deficits, with global developmental delay, and mild generalized dystonia. Figure 2 summarizes the historical and current case clinical informations.

## Discussion

This report documents the case of a toddler with anti-NMDAR encephalitis who was admitted for gastrointestinal symptoms. The subsequent acute onset of movement disorder symptoms in a previously healthy child was an urgent neurologic situation (2). Accurate diagnosis was essential because many conditions that induce motor symptoms are treatable, especially with early intervention. However, good outcomes often require an etiologically appropriate targeted approach (2).

The main cause of sudden-onset hyperkinetic movement in children is an acute dystonic reaction, often in response to anti-dopaminergic medications and antiemetics. In such cases, a dramatic improvement should be effected upon the withdrawal of the offending drug and the intravenous or intramuscular administration of an anticholinergic drug (3).

An important differential diagnosis in children exhibiting dystonia is GA-1 (OMIM no. 231670): a congenital condition caused by abnormal lysine metabolism due to a mutation in the glutaryl CoA-dehydrogenase (*GCDH*) gene on chromosome 19p13.2 (4). The resultant GCDH deficiency increases the concentrations of potentially neurotoxic metabolites. Indications of GA-1 include neonatal macrocephaly and frontoparietal brain atrophy that can be identified with neuroimaging. Affected children generally exhibit acute neurological deterioration between 6 and 18 months of age that may be triggered by a catabolic state caused by an infectious disease, surgery, or fever, including a severe febrile reaction to vaccination. Patients with GA-1 become acutely hypotonic, lose head control, and may exhibit abnormal movement as result of striatal injury. A metabolic diagnosis of GA-1 can be rendered based on a urine organic acid analysis; classically, glutaric acid levels remain elevated even when the patient is not in a catabolic state, and diagnosis of GA-1 can be confirmed by the identification of biallelic pathogenic *GCDH* variants (Table 1) (4, 5, 7).

**Table 1:** Comparison between an acute critical presentation of glutaric aciduria type I (GA-1) and anti-N-methyl-D-aspartate receptor (anti-NMDAR) encephalitis.

**Variable**	**GA-1**	**Anti-NMDAR encephalitis**
Age of onset	6–18 months	Any
Flu-like symptoms at onset	Often	Possible
Macrocephaly	Nearly always	Not associated
Developmental regression	Often	Possible^*^
Hyperkinesia	Often	Often
Seizures	Possible	Possible
CSF study	Normal	May involve pleocytosis, presence of oligoclonal bands and elevated CSF protein^**^
Brain MRI	Usually shows frontoparietal brain atrophy with widening of the Sylvian fissures; sometimes arachnoid cysts. May involve acute striatal necrosis^***^	Usually normal
Definitive diagnosis	DNA analysis or detection of 3-OH-glutaric acid with organic acid analysis	Presence of one or more of six major groups of symptoms and anti-GluN1 IgGs

*CSF, cerebrospinal fluid; MRI, magnetic resonance imaging; Ig, immunoglobulin*.

* *Children may present with development regression rather than psychosis*.

** *Pleocytosis range, 29–80%, normal CSF study does not exclude anti-NMDAR encephalitis*.

*** *Striatal/extrastriatal progression highly variable in form and speed*.

*References: (1, 4–6)*.

Encephalitis is a potentially lethal neurologic disease wherein inflammation of brain parenchyma leads to neurologic dysfunction (8). Although infectious agents are the main cause of encephalitis, cases of autoimmune encephalitis are increasingly being reported (8, 9). Since Dalmau et al. (10) described anti-NMDAR encephalitis in 2007, epidemiologic studies have implicated anti-NMDAR encephalitis as a predominant cause of autoimmune encephalitis, second only to demyelinating acute encephalitis (9).

Anti-NMDAR encephalitis is an immune-mediated disease associated with the production of IgGs that target the GluN1 subunit of NMDAR (11). The condition may induce a wide spectrum of autonomic, cognitive, and behavioral symptoms, including disturbed sleep and movement disorders (1, 6, 11). Anti-NMDAR encephalitis may present differently in children than in adults: e.g., toddlers may exhibit developmental regression as a predominant symptom, which should invoke clinical suspicion, especially if the regression has a sudden onset and is associated with a movement disorder (6). Other common symptoms exhibited by young children include insomnia, irritability, and seizures. Psychiatric disorders and dysautonomia are uncommon or less evident in very young children with anti-NMDAR encephalitis than in their adult counterparts (Table 1) (11, 12).

A diagnosis of anti-NMDAR encephalitis should be considered when patients develop four or more of the following symptoms within 3 months: cognitive-behavioral disturbance or psychiatric symptoms, language disorder, epileptic seizures, abnormal movements, dyskinesias, stiffness or dystonic postures, impaired consciousness, and autonomic dysfunction or central hypoventilation. In addition to excluding other disorders (e.g., altered mental status, extrapyramidal syndrome, and epilepsy), clinicians should perform electroencephalography to confirm anti-NMDAR encephalitis. Furthermore, CSF with pleocytosis or oligoclonal bands is suggestive of anti-NMDAR encephalitis (1, 6). A definitive diagnosis in patients exhibiting any of the aforementioned symptoms can be rendered upon the identification of IgG anti-GluN1 antibodies (Table 1) (1).

Alizapride is a metoxibenzamide drug that inhibits type 2 dopaminergic receptors (13). The use of anti-dopaminergic drugs, or any other typical or atypical antipsychotics, can worsen the clinical status or exacerbate the symptoms of patients with anti-NMDAR encephalitis—especially before immunomodulatory therapy has been initiated (14). Patients with anti-NMDAR encephalitis also appear to be at particular risk of experiencing adverse secondary effects of anti-dopaminergic medications, including drug-induced movement disorders (11).

Anti-NMDAR encephalitis should be treated as early in its course as possible with first line therapy (intravenous IVIg, high doses of steroids, plasmapheresis) (11). Although uncommon in the pediatric population, in the presence of teratoma or other malignancy, tumor removal results in substantial neurological improvement (6, 15). When first-line treatments fail, second-line immunotherapy is usually effective (15). Autoimmune encephalitis is not always monophasic; as relapses may occur, patients should be enrolled in long-term maintenance therapy to maximize therapeutic gains and the patient's ultimate functional state. Monthly IVIg infusion or plasmapheresis with the sustained administration of oral corticosteroids or a steroid-sparing agent may be considered for patients with stalled recovery. As a well-established standard regarding duration of maintenance-IVIg therapy remains lacking, clinicians need to decide the duration based on the evolution of the patient's status (11).

The acquired macrocephaly and prominence of CSF spaces identified in the present case (Figure 1) first misleading indicators of a metabolic disease, were attributable to the benign enlargement of the subarachnoid spaces (BESS). BESS, also known as external hydrocephalus, and constitutional megalencephaly are the major causes of macrocephaly in infants (16). It is transient and characterized by disproportionate skull and brain volumes, which is radiologically evidenced by the widening of the bifrontal and anterior interhemispheric CSF spaces. BESS does not require specific treatment: as the child grows older, the subarachnoid space fluid collection will resolve or become minimal (16). Children with BESS generally follow neurological developmental trajectories, and BESS may only become evident when an underlying disease triggers a clinical investigation. Macrocephaly was unrelated to anti-NMDAR encephalitis playing a role as a confusing factor in the etiological investigation.

Importantly, our patient had a good outcome owing to diagnostic suspicion and early treatment. The present case highlights the importance of considering autoimmune encephalitis, a treatable disease whose prognosis depends on early diagnosis and treatment, as a differential diagnosis of acute-onset movement disorders because it rarely follows an established clinical course and may mimic those of several other conditions. While our report is limited by its isolation in the literature, which limited the evaluation of the efficacy of our treatment method, it contributes to the ongoing elucidation of the condition's clinical manifestation and treatment (12)—particularly in pediatric cases.

## Patient Perspective

The patient remains in clinical follow-up. The family has reported perceiving a great improvement in symptoms and quality of life with respect to the initial condition.

## Ethics Statement

The studies involving human participants were reviewed and approved by The Hospital Pequeno Principe's Ethics Research Committee reviewed and approved the study (CAAE: 33349020.6.0000.0097). The authors obtained written informed consent from the parents of the patient. Written informed consent to participate in this study was provided by the participants' legal guardian/next of kin. Written informed consent was obtained from the individual(s) for the publication of any potentially identifiable images or data included in this article.

## Author Contributions

DV participated in the clinical management, conceptualized the study, drafted the initial manuscript, and reviewed and revised the manuscript. MS reviewed and revised the manuscript. MZ participated in the clinical management, and reviewed and revised the manuscript. MC coordinated the study, revised the manuscript, and conducted the critical reviews of the manuscript for key intellectual content. All authors approved the final manuscript and agree to be accountable for all aspects of the work.

## Conflict of Interest

The authors declare that the research was conducted in the absence of any commercial or financial relationships that could be construed as a potential conflict of interest.

## Abbreviations

BESS: benign enlargement of the subarachnoid spaces
CSF: cerebrospinal fluid
GA-1: glutaric aciduria type 1
GCDH: glutaryl CoA-dehydrogenase
Ig: immunoglobulin
MRI: magnetic resonance imaging
NMDAR: N-methyl-D-aspartate receptor.
